# An Early Holiday Surprise: Cholecystitis Wrapped in Takotsubo Cardiomyopathy

**DOI:** 10.5811/cpcem.2020.1.45474

**Published:** 2020-03-02

**Authors:** Kevin Gould, Stephen Miller, Joel Moll

**Affiliations:** Virginia Commonwealth University, Department of Emergency Medicine, Richmond, Virginia

## Abstract

This is a novel case report of a 44-year-old woman who presented to the emergency department with epigastric pain wrapping around to her back. She had no risk factors for cardiac disease, but her initial electrocardiogram (ECG) showed a Wellens syndrome pattern and she was taken urgently to the catheterization lab. After a negative catheterization, she underwent cardiac magnetic resonance imaging, which was positive for Takotsubo cardiomyopathy (TC). Ultimately, abdominal computed tomography revealed that she had cholecystitis, which likely was the cause of her TC and ECG changes.

## INTRODUCTION

Takotsubo cardiomyopathy (TC), also known as stress cardiomyopathy, is a rare but dangerous condition that can be encountered in the emergency department (ED). TC can be difficult to diagnose as it can present as chest pain or abdominal pain; it is often identified after the fact by way of cardiac magnetic resonance imaging. There are known electrocardiographic (ECG) changes as well as transthoracic echocardiogram findings, but these can be subtle.^1^,^2^ We present a case report of a middle-aged female who presented to the ED with abdominal pain and was found to have concerning changes on her ECG.

## CASE REPORT

A 44-year-old female with past medical history significant for gastroesophageal reflux disease, depression, insomnia, and cervical cancer in remission presented in mid-December via private vehicle to the ED with epigastric pain since the prior evening. The patient had attempted treatment at home with over-the-counter antacids without relief. She described her pain as epigastric burning, wrapping around her chest to her back, with associated diaphoresis, nausea, and vomiting. She denied fevers, hematemesis, dysuria, or vaginal discharge. The patient’s cervical cancer had been treated with total abdominal hysterectomy, chemotherapy, and radiation. Her only daily medication was hormone replacement therapy. She reported drinking one alcoholic beverage per day but denied tobacco or drug use. Family history was notable for coronary artery disease in her mother, who died of a myocardial infarction at age 54.

**Image 1:**
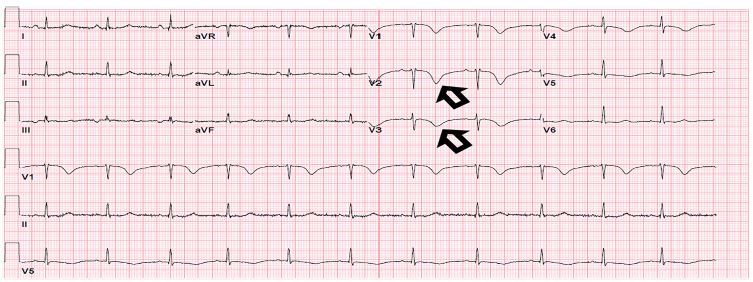
Initial electrocardiogram with Wellen’s syndrome morphology.

The patient’s vital signs in triage were as follows: heart rate 77 beats per minutes; blood pressure 150/90 millimeters of mercury; respiration rate 15 breaths per minute; temperature: 36.8º Celsius; and oxygen saturation 98%. Pain was 9/10 (epigastric). An ECG was immediately obtained (Image 1). The ECG showed possible ischemia due to deep t-wave inversions across leads V1–4, and the patient was placed on a cardiac monitor.

**Image 2:**
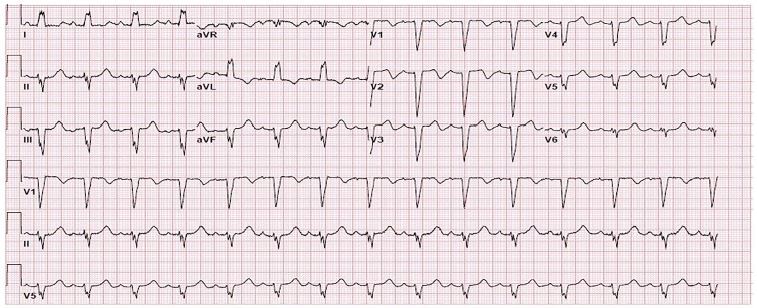
Electrocardiogram approximately one hour after arrival showing new left bundle branch block.

The patient’s physical exam revealed marked right upper quadrant and epigastric tenderness with a positive Murphy’s sign. Her initial labs showed mild hyponatremia (133 milligrams [mg] per deciliter), leukocytosis (18.6 thousand) and troponin-I of 0.18 (normal range: < 0.03 – 0.39 nanograms per milliliter Cardiology was urgently consulted and a stat bedside transthoracic echo showed septal wall motion abnormalities, possible apical hypokinesis, and an estimated left ventricular ejection fraction (LVEF) of 45–50%. Repeat ECG during cardiology evaluation (Image 2) showed a new left bundle branch block (LBBB). Due to the new LBBB and t-wave inversions on initial ECG concerning for Wellens syndrome the patient was given 324 mg aspirin, 4000 units heparin, 180 mg ticagrelor, and emergently taken to the catherization lab.

A left heart catheterization was performed and revealed no significant coronary artery disease. Upon admission to the cardiac intensive care unit (ICU), the patient continued to complain of epigastric pain for which she received acetaminophen/oxycodone, pantoprazole, and a gastrointestinal (GI) cocktail that helped relieve her pain. Chest and abdominal plain radiographs obtained shortly thereafter did not show any acute pathology.

That evening, the patient underwent computed tomography abdomen/pelvis with intravenous (IV) contrast, which demonstrated gallbladder wall thickening with gallstones in both the gallbladder and cystic duct. The patient was started on empiric IV piperacillin-tazobactam. Gastroenterology and general surgery were consulted. Overnight the patient remained afebrile and her pain fully resolved.

**Image 3:**
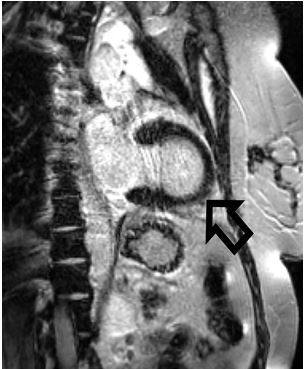
Sagittal magnetic resonance imaging showing apical ballooning of left ventricle.

To help elucidate the cause of the patient’s wall motion abnormalities, a cardiac magnetic resonance imaging (MRI) was performed the following day. The MRI was significant for apical ballooning of the left ventricle with an ejection fraction of 49% (Image 3). Based on these findings the patient was diagnosed with Takotsubo cardiomyopathy.

The patient was transferred from the cardiac ICU to the floor, and elected conservative management of her cholecystitis via dietary changes and surgical follow-up. At discharge on hospital day 3, she was started on metoprolol 25 mg twice daily and lisinopril 5 mg daily for blood pressure control and cardiac remodeling protection. She underwent an outpatient laparoscopic cholecystectomy approximately two months later. At her three-month cardiology follow-up, the patient had persistent LBBB and her transthoracic echo showed LVEF of 55–60% with paradoxical septal wall motion, but there was no apical ballooning. At six months post-diagnosis, she had had no further episodes of TC.

**CPC-EM Capsule**

What do we already know about this clinical entity?

Takotsubo cardiomyopathy (TC) can present in concert with a variety of other conditions and its presentation mimics acute coronary syndrome.

What makes this presentation of disease reportable?

TC can present in a myriad of ways, in this case, due to cholecystitis. If TC is missed, it could lead to hemodynamic compromise and a potentially bad outcome.

What is the major learning point?

Cholecystitis can cause TC.

How might this improve emergency medicine practice?

Providers should keep TC on the differential for patients presenting with chest pain, as well as those presenting with abdominal pain.

## DISCUSSION

The patient’s chief complaint of epigastric pain has a wide differential that includes both abdominal and thoracic etiologies. Differential diagnoses include esophagitis, esophageal spasm, gastroesophageal reflux, gastritis, peptic ulcer disease, biliary colic, cholecystitis, cholangitis, esophageal rupture, pancreatitis and, because many of these conditions can mimic acute coronary syndrome (ACS), a consideration of myocardial ischemia is warranted. A meta-analysis of ACS presentations to both primary care and the ED found that for those presenting with epigastric pain, there was a 91% specificity (95% confidence interval [CI], 85.0–95.4) but only a 5% sensitivity (95% CI, 2.1–10.8)^3^ with relation to acute coronary ischemia. This, coupled with the National Registry of Myocardial Infarction II study, which found that 33% of patients with acute myocardial ischemia present without chest pain,^4^ places ACS in the differential for any patient who presents with GI symptoms. Additionally, women with acute coronary ischemia are more likely to present with atypical, non-chest pain and are at a higher risk of discharge from the ED, especially if they are <55 years old (odds ratio [OR] [6.7], 95% CI, 1.3–32.5).^5^

The patient’s initial ECG was concerning for Wellens syndrome, a condition most commonly associated with critical stenosis of the left anterior descending (LAD) artery and increased risk of anterior wall myocardial infarction if not treated with percutaneous coronary intervention. Wellens syndrome can present with one of two t-wave abnormalities: a biphasic T-wave (type A, sometimes referred to as a “saddle wave,” occurs in 25% of cases) and a deeply inverted T-wave (type B, occurs in 75% of cases).^6^ This change happens in V2–3 but may extend to V1–6. Wellens types A and B can be seen as T-wave changes along a continuum, as initial LAD occlusion will lead to type A changes and reperfusion can lead to type B changes. These changes can occur in a “stuttering” format if the LAD occlusion causes intermittent ischemia, with reoccurrences of type A and B morphologies.^6^ Wellens syndrome can also manifest due to vasospasm when no LAD stenosis is present.

TC is the cause of 1–2% of ACS presentations to the ED.^1^ The prevalence of TC in the US is estimated to be 5.2 per 100,000 women and 0.6 per 100,000 men.^7^ First investigated by Sato et al in 1990 in Japan, the entity of Takotsubo, or stress cardiomyopathy, was named due to the apical ballooning of the heart, which looks similar to a pot used to catch octopi.^8^ Often referred to as “broken heart syndrome,” it has been reported in post-menopausal women who have recently suffered severe emotional stress. Women >55 years old had 4.8 times higher odds for developing TC compared to those <55 years old.^7^

TC diagnostic criteria include the following: 1) new concerning ECG changes (most commonly ST-segment elevation and/or T-wave inversions) or increased troponins (1.8× increase as opposed to the average 6× increase associated with myocardial infarction); 2) transient akinesis /dyskinesis of the left ventricle (typically apical); and 3) absence of >50% coronary artery stenosis or culprit lesion.^1^,^2^ While TC has been traditionally diagnosed via cardiac MRI, bedside transthoracic echocardiogram can be used to help diagnose it by noting apical ballooning that crosses several areas of cardiac perfusion when using the apical 4-chamber view.^9^

Many etiologies have been proposed, most relating to catecholamine-induced vasospasm, and coronary artery spasm.^10^ Left ventricular outflow tract (LVOT) obstruction and estrogen deficiency are additional risk factors for the development of TC.^11^ Most TC patients will be hemodynamically stable upon presentation but approximately 10% will present in cardiogenic shock.^12^ Factors associated with a shock presentation include atrial fibrillation (OR [2.03]; 95% CI, 1.22–3.40; P = 0.007), left ventricular ejection fraction <45% (OR [2.49]; 95% CI, 1.63–3.80; P <0.001), and catecholamine release (OR [2.84]; 95% CI, 1.96–4.12; P <0.001).^12^ LVOT obstruction is associated with 19% of TC-induced cardiogenic shock.^13^ While there does not appear to be any increased mortality related to LVOT obstruction-related cardiogenic shock, the management is very different.^12^

Absence of LVOT obstruction is treated with cardiogenic inotropes (typically dobutamine) to increase cardiac output. Presence of LVOT obstruction is treated with fluid resuscitation to improve pre-load as long as pulmonary congestion is not present, along with beta-blocker therapy to improve hemodynamics and potentially resolve the obstruction.^13^ It is, therefore, imperative that any patients in cardiogenic shock undergo stat echocardiogram to assess for LVOT obstruction as this will radically change their management.

In our case report, the patient’s cholecystitis and the associated catecholamine surge due to pain likely caused her TC, which resulted in her ECG changes. While case reports have linked pancreatitis and concomitant Wellens syndrome with TC, our case is novel in that our patient presented with TC and Wellens syndrome due to acute cholecystitis.^14^

## CONCLUSION

Takotsubo cardiomyopathy can mimic the presentation of acute coronary syndrome and will often meet criteria for immediate catheterization. This case reports a type B Wellens-like ECG pattern and development of a new LBBB with related TC from acute cholecystitis. Emergency providers need to be aware of TC as it can present with or quickly develop into cardiogenic shock whose treatment hinges on the presence of LVOT obstruction. While cardiac MRI is still the gold standard for diagnosis of TC, bedside echocardiography can give the provider crucial information on the patient’s cardiac hemodynamics, helping direct the best management of TC until the patient can be transported to the catheterization lab or the cardiac ICU. Importantly, as in this case, the underlying cause of TC must also be found and managed, along with the TC itself.
